# The role of social risk factors and engagement with maternity services in ethnic disparities in maternal mortality: A retrospective case note review

**DOI:** 10.1016/j.eclinm.2022.101587

**Published:** 2022-07-29

**Authors:** Eleanor Cosstick, Rachel Nirmal, Fiona Cross-Sudworth, Marian Knight, Sara Kenyon

**Affiliations:** a University of Birmingham Medical School, Birmingham B15 2TH, UK; b Institute of Applied Healthcare, University of Birmingham, Birmingham B15 2TT, UK; c National Perinatal Epidemiology Unit, Nuffield Department of Population Health, University of Oxford, Oxford OX3 7LF, UK

**Keywords:** Ethnic minorities, Maternal death, Risk factors, Access to maternity services, Interpreter services, Maternal co-morbidities

## Abstract

**Background:**

Reasons for ethnic disparities in maternal death in the UK are unclear and may be explained by differences in social risk factors and engagement with maternity services.

**Methods:**

In this retrospective systematic case note review, we used anonymised medical records from MBRRACE-UK for all Other than White, and White European/Other women plus a random sample of White British/Irish women who died in pregnancy or up to 1 year afterwards from 01/01/2015 to 12/31/2017. We used a standardised data extraction tool developed from a scoping review to explore social risk factors and engagement with maternity services.

**Findings:**

Of 489 women identified, 219 were eligible for the study and 196 case notes were reviewed, including 103/119 from Other than White groups, 33/37 White European/Other and a random sample of 60/333 White British/Irish. The presence of three or more social risk factors was 11⋅7% (12/103) in Other than White women, 18⋅2% (6/33) for White European/Other women and 36⋅7% (22/60) in White British/Irish women. Across all groups engagement with maternity services was good with 85⋅5% (148/196) receiving the recommended number of antenatal appointments as was completion of antenatal mental health assessment (123/173, 71⋅1%). 15⋅5% (16/103) of Other than White groups had pre-existing co-morbidities and 51⋅1% (47/92) had previous pregnancy problems while women across White ethnic groups had 3⋅2% (3/93) and 33⋅3% (27/81) respectively. Three or more unscheduled healthcare attendances occurred in 60⋅0% (36/60) of White British/Irish, 39⋅4% (13/33) in White European/Other and 35⋅9% (37/103) of Other than White women. Evidence of barriers to following healthcare advice was identified for a fifth of all women. None of the 17 women who required an interpreter received appropriate provision at all key points throughout their maternity care.

**Interpretation:**

Neither increased social risk factors or barriers to engagement with maternity services appear to underlie disparities in maternal mortality. Management of complex social factors and interpreter services need improvement.

**Funding:**

National Institute for Health Research (NIHR) Applied Research Collaboration West Midlands.

**Research in context**

**Evidence before this study**

MBRRACE-UK Confidential Enquiries, which utilise tried and tested methodology of multidisciplinary groups of senior clinicians systematically reviewing a defined set of deaths to assess care quality and support future practice, additionally identified that British Black women are four times more likely, and Asian women twice as likely, to die during or after pregnancy than White women. This is similar to other high-income country findings. Other than White women have also sometimes received a poorer quality of care than White women. While reasons for maternal mortality disparity are unclear, issues with lack of access or engagement with maternity services, such as late booking and not attending appointments, as well as multiple physical, obstetric or social risk factors have been linked to higher mortality rates.

**Added value of this study**

This study was a retrospective systematic review of medical records of a sample of the women who died 2015–2017 in the UK using a data extraction tool developed from a scoping review. While review of medical records has limitations, neither increased social risk factors or access and engagement with maternity services appear to underlie ethic disparities in maternal death rates. These factors appear to be most frequently seen in White British women.

**Implications of all the available evidence**

Management of complex social factors and provision of interpreter services are targets for improvement and could potentially reduce maternal deaths across all ethnic groups. Further research into reasons for the disparity in ethnicity and maternal mortality is urgently needed, and should focus on how best to address clinical, social and cultural complexity and providing individualised care.

Alt-text: Unlabelled box

## Introduction

A maternal death is conventionally defined as a death of a woman during or within 42 days of the end of pregnancy due to an associated or exacerbated cause.^1^ The significance of late maternal deaths, occurring after 42 days but within a year following pregnancy, is increasingly recognised and being investigated.^2^^,^^3^

Maternal deaths in high income countries, including the UK, are uncommon.^4^^,^^5^ Recent figures indicate a maternal mortality rate of 8.8 per 100,000 maternities in the UK.^6^ The most recent Confidential Enquiry reviewed all maternal deaths from 2017 to 2019 and reported that Black women in the UK were four times more likely, and Asian women almost twice as likely, to die during or shortly after pregnancy than white women.^6^ Similar ethnic differences in maternal mortality rates have been reported over a number of years yet the reasons for such disparities remain unclear.^7^

There is some evidence to suggest that access to and engagement with maternity services is problematic for ethnic minority women in the UK, and that Other than White groups are more likely to access antenatal care late.^8^^,^^9^ However, the causes and prevalence of such barriers amongst women who died is not known.

The MBRRACE-UK report published in 2020 reported that 90% of women who died during or within a year after pregnancy experienced a “constellation of biases”.^6^ These include having physical and mental health problems, delayed antenatal care, being non-English speaking, and complex social factors such as domestic abuse, smoking, and unemployment. All of these factors directly relate to or can affect access and engagement with maternity services across ethnic groups.^6^

Uniquely, this study aimed to systemically review the anonymised medical records of a sample of women who died, during or within one year of pregnancy, between 2015 and 2017, to explore whether social risk factors and barriers to access and engagement with maternity services as documented within maternal case notes could underlie these disparities in maternal mortality.

## Methods

An initial literature scope identified access and engagement barriers faced by Other than White groups using maternity services in the UK. This informed the development of a standardised data extraction tool to systematically review anonymised notes of women from Other than White groups, White British/Irish and White European/Other women, who died during or up to a year following pregnancy in the UK from 2015-2017.

### Literature review

We searched MEDLINE and CINAHL databases using a search strategy developed from key words and synonyms for Black or Asian, maternity care and access (see Supplementary Table S1). Searches were limited to UK, peer-reviewed, English-language articles published from 01 01 2010 to 06 02 2020.

All article types were included as the search aims were explorative. Relevant articles included an outcome of access or engagement with maternity services and analysis or sub-group analysis for a Black or Asian population, or a population made up of a Black or Asian majority. Two independent researchers screened titles, abstracts and full texts against pre-defined eligibility criteria (see Supplementary Table S2) with queries resolved by discussions involving a third researcher.

Initial MEDLINE and CINAHL searches obtained 55 articles. Following removal of duplicates and screening of results, 18 articles remained eligible for review. The full texts for two articles were unavailable so a total of 16 articles were reviewed. The stages and reasons for exclusion plus study characteristics and relevant findings of included articles into Supplementary Tables are both recorded in supporting information (see Supplementary Figure 1 and Table S3).

Of 16 included articles, half were qualitative studies with interview or focus group methodologies, six retrospective analyses of survey-based or routinely collected data, one Q methodology study and one review article. Issues identified included language barriers, problems with interpreters, cultural barriers, and inadequate knowledge of maternity services (from not being UK citizens), as well as complex social, physical and mental health needs, which were inadequately addressed.^10^, ^11^, ^12^, ^13^, ^14^, ^15^, ^16^, ^17^, ^18^, ^19^, ^20^, ^21^, ^22^, ^23^ A number of factors contributed to later booking, fewer appointments and dissatisfaction with care amongst Black and Asian women.^10^^,^^12^^,^^13^^,^^15^^,^^17^, ^18^, ^19^, ^20^, ^21^, ^22^^,^^24^^,^^25^ A summary of the key literature findings is included in Supplementary Table S4.

### Development of the standardised data extraction tool

Literature review findings informed the majority of items in the data extraction tool. Input was also obtained through screening of relevant guidelines. Complex social factors (detailed in Box 1), as defined by the 2018 Revolving Doors Agency and Birth Complications report on the perinatal experiences of women facing multiple disadvantage (defined as three or more complex social factors), were also included.^26^ Following piloting and minor revisions, the final version of the data extraction form can be seen in Supplementary Table S5.

Box 1 **Complex social risk factors**

**Table:** 

Revolving Doors Agency and Birth Campanions^26^ report list of complex social factors of multiple disadvantage	
• Domestic violence or abuse	• Physical disability
• Substance misuse	• Learning difficulty
• Mental health issues	• Significant financial need
• Criminal justice involvement	• Recent migrant (less than 1 year in UK)
• Homelessness	• Unable to speak or understand English
• Young age (under 20 years)	• Social services involvement

Alt-text: Unlabelled box

### Medical record review

Anonymised medical records for women who died during or within one year following pregnancy from 2015 to 2017 (inclusive) were examined. We included the complete cohort from Other than White groups (*n =* 119) and reviewed case notes from 103 women, including those of mixed Black or Asian ethnicities. We included the complete cohort of White European/Other women (*n =* 37) who died as they may face language barriers and not be familiar with the UK health system.^27^ and reviewed case notes from 33. The remaining 60 White women who died and whose records were examined were randomly sampled using a computer-based random number generator from the 333 White British/Irish maternal deaths (2015–2017), making this the largest group of a single ethnicity.

These records were used to collect data on items included in the standardised extraction form for each woman. Two independent researchers reviewed the medical records and extracted data for each woman who died. Queries regarding interpretation of medical records were resolved by discussions, re-examination of records and consultation with a third reviewer.

### Analysis

Data for each item on the extraction tool from all cases were entered into an Excel spreadsheet. Frequencies and percentages were calculated for background characteristics of the women who died, features of their deaths and the care they received.

Women's ethnicities were grouped according to UK census categorisation.^28^ In line with guidance on writing about ethnicity, instead of using the terms Black, Asian and other Ethnic minority (BAME/BME) we refer to these women as from Other than White groups.^29^ Comorbidities (such as cardiac conditions, epilepsy, cancer) or previous pregnancy problems (such as intra-uterine death, post-partum haemorrhage, gestational diabetes) were considered significant if they required referral for obstetrician-led care.

Social risk factor information was identified by healthcare professionals’ documentation in the case notes such as tickbox checklists, appointment summaries or letters. This information usually originated from women often at the initial booking into maternity care appointment as part of a routine assessment but may also have been added to or amended at later points. Complex social factors were defined using the Revolving Doors Agency and Birth Complications report described in Box 1.^26^ These were deemed appropriately addressed where they were identified and discussed or referred to support services by maternity care providers.

Late booking was defined according to the NHS key performance indicator recommending that antenatal assessment should occur before 13 weeks.^30^ Whether or not women received the minimum number of recommended antenatal visits was determined by comparing the number of routine visits to the National Institute of Health and Care Excellence (NICE) antenatal care guidance (ten for nulliparous and seven for multiparous women),^31^ taking into account gestational age at birth and death. It was not possible to objectively quantify the number of women who received a minimum standard of postnatal care as NICE do not set out a standard schedule for postnatal visits but recommend local planning and individualised care strategies.^32^ Screening was defined as routine NHS recommended blood tests and/or ultrasound scans for both maternal and fetal conditions.^33^ Routine mental health assessment should be undertaken in both the antenatal and postnatal periods and current NICE guidelines suggest using the Whooley questions.^34^

### Details of ethics approval

Identifiable MBRRACE-UK data were collected in England and Wales without consent with approval of the Secretary of State for Health and Social Care under Section 251 of the NHS Act 2006 (15/CAG/0119). Data were collected in Scotland without consent with approval from the Public Benefit and Privacy Panel for Health and Social Care (1920–0131). Identifiable information was not provided from Northern Ireland. The legal basis for this activity is Article 6 (1)(e) and Article 9 (2)(i) under the General Data Protection Regulation. All MBRRACE-UK information was anonymised prior to use for research.

Permission to access anonymised MBRRACE-UK data was obtained from the Healthcare Quality Improvement Partnership (HQIP) for the purpose of this study. Ethical approval for the study was granted by the University of Birmingham Internal Research Ethics Committee (IREC) on 29.01.2020 (Reference: IREC2019/ 1646192) and 25.01.2021 (Reference: IREC2020/1762770).

### Role of the funding source

The funder of the study had no role in study design, data collection, data analysis, data interpretation, or writing of the report. All authors had access to the data and accept responsibility for the decision to submit for publication.

## Results

### Maternal characteristics

**Figure 1:**
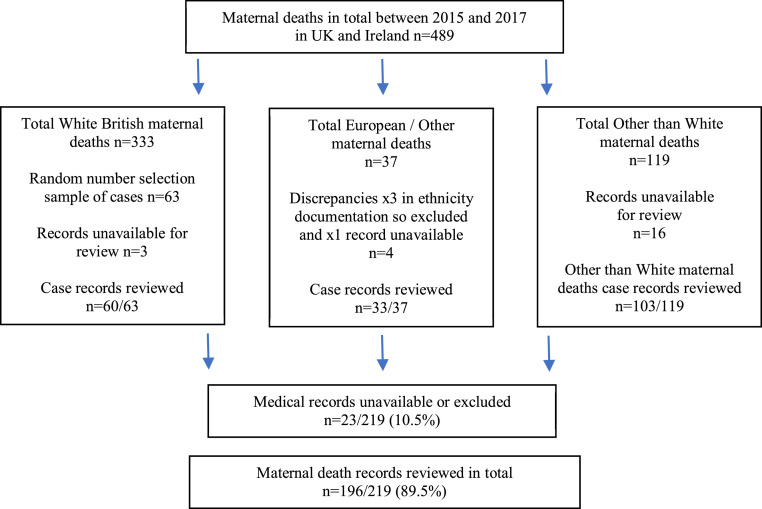
Flow-chart of women who died in 2015-2017 per ethnic group and those whose medical records were reviewed.

Of the 219 maternal deaths, medical records were unavailable for 20 women. These were records of women who died in the late postnatal period whose relevant records were not able to be obtained from hospitals by MBRRACE UK. Three of the women in the White European/Other group had been incorrectly classified and were also excluded making a total of 23/219 (10⋅5%). Thus, our final sample comprised 103/119 women from Other than White groups and 93 White women, of whom 33/37 were White European/Other and 60/63 were White British/Irish (see Figure 1 for details).

Black African was the commonest ethnicity amongst Black women (see Table 1 for maternal characteristics). Pakistani was the commonest ethnicity among Asian women. The majority of women from Other than White groups (63/103, 61⋅2%) were UK citizens, although citizenship was unknown for over a tenth (14/103, 13⋅6%). Most White European/Other women were European Union (EU) Citizens (24/33, 72⋅7%).

**Table 1:** Maternal characteristics.

			WhiteBritish/Irish women *n =* 60 (%)^a^	European/Other women *n =* 33 (%)^a^	Other thanWhite women *n =* 103 (%)^a^
**Ethnicity**	Black or Black British	Caribbean	N/A	N/A	11 (10.7)
		African	N/A	N/A	33 (32)
	Asian	India	N/A	N/A	14 (13.6)
		Pakistani	N/A	N/A	20 (19.4)
		Bangladeshi	N/A	N/A	4 (3.9)
		Chinese	N/A	N/A	4 (3.9)
		Other Asian Background	N/A	N/A	5 (4.9)
	White	British/Irish	60 (100)	N/A	N/A
		Other	N/A	33 (100)	N/A
	Mixed	White & Black Caribbean	N/A	N/A	4 (3.9)
		White & Black African	N/A	N/A	3 (2.9)
		White & Asian	N/A	N/A	0 (0)
		Other mixed background	N/A	N/A	3 (2.9)
	Unknown		N/A	N/A	2 (1.9)
**Citizenship status**	UK Citizen		60 (100)	8 (24.2)	63 (61.2)
	EU Citizen		0 (0)	24 (72.7)	3
	Non-EU Citizen		0 (0)	1 (3)	6 (5.8)
	Asylum seeker/refugee/undocumented migrant		0 (0)	0 (0)	5 (4.9%)
	Student, work or spousal visas/indefinite leave to remain		0 (0)	0 (0)	12 (11.7)
	Unknown		0 (0)	0 (0)	14 (13.6)
**Age at death (years)**	29 and under		28 (46.7)	7 (21.2)	33 (32)
	30 to 39		28 (46.7)	22 (66.7)	56 (54.4)
	40 and over		4 (6.7)	4 (12.1)	14 (13.6)
**Parity**	Nulliparous		27 (45)	13 (39.4)	34 (33)
	Multiparous		31 (51.7)	19 (57.6)	68 (66.0)
	Unknown		2 (3.3)	1 (3)	1 (1)
**BMI (kg/m^2^)**	24.9 or less		22 (36.7)	19 (57.6)	35 (34)
	25 to 29.9		11 (18.3)	5 (15.2)	32 (31.1)
	30 to 34.9		11 (18.3)	3 (9.1)	18 (17.5)
	35 and over		12 (20)	4 (12.1)	17 (16.5)
	Unknown		4 (6.7)	2 (6.1)	1 (1)
**Occupation**	Employed		26 (43.3)	20 (60.6)	53 (51.5)
	Unemployed		21 (35)	6 (18.2)	19 (18.4)
	Housewife		8 (13.3)	5 (15.2)	22 (21.4)
	Unknown/other		5 (8.3)	2 (6.1)	9 (8.7)
**Smoking**			29 (48.3)	8 (24.2)	6 (5.8)

a Due to rounding percentages may not add to 100%.

The majority of women from Other than White groups were multiparous (68/103, 66⋅0%). Obesity (BMI ≥ 30kg/m^2^) was identified in 38⋅3% (23/60) of the White British/Irish women, 34⋅0% (35/103) of Other than White and 21⋅2% (7/33) of White European/Other women. Over a third (21/60, 35⋅0%) of White British/Irish women were unemployed, while this was 18⋅4% (19/103) in Other than White groups and 18⋅2% (6/33) in White European/Other women.

Smoking was identified in 6/103 (5⋅8%) women from Other than White groups while for White British/Irish women who died it was 48⋅3% (29/60).

There was 15⋅5% (16/103) of Other than White women with three or more physical comorbidities and 51⋅1% (47/92) had previous pregnancy problems while women in White ethnic groups had 3⋅2% (3/93) and 33⋅3% (27/81) respectively (see Tables 2 and 3).

**Table 2:** Pre-existing physical comorbidities and selected risk factors.

	White British/Irish women *n =* 60 (%)	White European/Other women *n =* 33 (%)	Other than White women *n =* 103 (%)
**Number of women with any pre-existing physical comorbidity, BMI >30 or age over 40**			
None	21 (35.0%)	13 (39.4%)	32 (31.1%)
One	24 (40.0%)	10 (30.3%)	37 (35.9%)
Two	13 (21.7%)	9 (27.3%)	18 (17.5%)
Three or more	2 (3.3%)	1 (3.0%)	16 (15.5%)
**Number of women with any pre-existing physical comorbidity (excluding BMI and age >40)**			
None	36 (60.0%)	19 (57.6%)	47 (45.6%)
One	19 (31.7%)	9 (27.3%)	38 (36.9%)
Two	5 (8.3%)	4 (12.1%)	10 (9.7%)
Three or more	0	1 (3.0%)	8 (7.8%)

**Table 3:** Previous pregnancy problems.

	WhiteBritish/Irish women*n =* 51 (%)^a^	White European/Other women *n =* 30 (%)^a^	Other than White women *n =* 92 (%)^a^
Number of women with previous pregnancy problems	15 (29.4%)	12 (40%)	47 (51.1%)
**Previous pregnancy problems**			
Caesarean-section	9 (17.6%)	4 (13.3%)	25 (27.2%)
Post-partum haemorrhage	1 (2.0%)	3 (10.0%)	4 (4.3%)
Pre-eclampsia / pregnancy induced hypertension	2 (3.9%)	2 (6.7%)	13 (14.1%)
Miscarriage (3+) / ectopic	0	0	6 (6.5%)
Stillbirth / neonatal death	1 (2.0%)	2 (6.7%)	3 (3.3%)
Intrauterine growth restriction / small for gestational age / oligohydramnios	4 (7.8%)	2 (6.7%)	11 (12.0%)
Gestational diabetes mellitus	1 (2.0%)	0	3 (3.3%)
Puerperal / peri-partum psychosis / postnatal depression	2 (3.9%)	1 (3.3%)	2 (2.2%)
Placental abruption / previa / antepartum haemorrhage	0	0	5 (5.4%)

a Due to rounding percentages may not add to 100%.

### Complex social factors

Multiple disadvantage (three or more complex social factors) was overall faced by a fifth of women: 36⋅7% (22/60) of White British/Irish women, 18⋅2% (6/33) of White European/Other women and 11⋅7% (12/103) of women in the Other than White group (see Table 4). Of women in the Other than White group 55⋅3% (57/103) had no complex social factors compared to 36⋅7% (22/60) of White British/Irish women and 36⋅4% (12/33) of White European/Other women. There was 15⋅2% (5/33) of recent migrants in the White European/Other group while Other than White group had 3⋅9% (4/103).

**Table 4:** Frequency of complex social risk factors.

	White British/Irish women *n =* 60 (%)^a^	European/Other women *n =* 33 (%)^a^	Other than White women *n =* 103 (%)^a^
Significant financial need	4 (6.7)	4 (12.1)	6 (5.8)
Insecure Housing^b^	7 (11.7)	0 (0)	13 (12.6)
Substance misuse	21 (35)	4 (12.1)	4 (3.9)
Criminal justice involvement	13 (21.7)	2 (6.1)	0 (0)
Social services involvement	23 (38.3)	4 (12.1)	13 (12.6)
Learning disability	4 (6.7)	0 (0)	0 (0)
Physical disability	2 (3.3)	1 (3)	7 (6.8)
Domestic abuse	18 (30)	3 (9.1)	5 (4.9)
Mental health issues	29 (48.3)	11 (33.3)	24 (23.3)
Young (<20 years)	5 (8.3)	0 (0)	2 (1.9)
Recent migrant (<1 year)	0 (0)	5 (15.2)	4 (3.9)
Does not speak/understand English	0 (0)	10 (30.3)	7 (6.8)
**Number of women with complex social risk factors**			
None	22 (36.7)	12 (36.4)	57 (55.3)
One	8 (13.3)	10 (30.3)	26 (25.2)
Two	8 (13.3)	5 (15.2)	8 (7.8)
Three or more	22 (36.7)	6 (18.2)	12 (11.7)

a Due to rounding percentages may not add to 100%.

b We have used insecure housing instead of homeless to reflect the findings of our literature search.

Existing complex social factors were identified by maternity services among women from the Other than White group in 66/73 (90⋅4%) (see Table 5). A similar number of both Other than White and White British women had social risk factors both identified and actioned, such as referring to specialist services, by maternity services (46/73, 63⋅0% and 64/102, 62⋅7% respectively) while for White European/Other women, 77⋅5% (31/40) were identified and a quarter (10/40, 25⋅0%) were addressed.

**Table 5:** Complex social factors identified and addressed for women booked by maternity services.

	White British/Irish women *n =* 51 (%)	White European/Other women *n =* 30 (%)	Other thanWhite women *n =* 92 (%)
Total complex social factors identified amongst those who received any antenatal care	102	40	73
Number (%) of social factors identified by maternity services	84/102	31/40	66/73
	(82.4%)	(77.5%)	(90.4%)
Number (%) of social risk factors identified and addressed by maternity services	64/102	10/40	46/73
	(62.7%)	(25.0%	(63.0%)

### Features of maternity care

A quarter of women (43/173, 24⋅9%) attended their first antenatal visit late (after 13 weeks gestation) across the cohort (see Table 6). Among the White European/Other group this was a third (9/30, 30⋅0%), while for Other than White groups, it was a fifth (20/92, 21⋅7%). A fifth of both women from Other than White (19/92, 20⋅7%) and White British/Irish groups (10/51, 19⋅6%) declined screening. Overall, most women had the recommended number of antenatal visits for their gestation, and nearly all antenatal non-attendances were followed up. Fewer than half of women from Other than White groups received three or more postnatal visits (14/32, 48⋅3%). Over three quarters of eligible women in both White groups received three or more postnatal visits: 15/18 (83⋅3%) in White European/Other women and 48⋅3% (14/29) of Other than White women.

**Table 6:** Features of antenatal and postnatal care.

	White British/Irish women *n =* 60 (%)^a^	European/Other women *n =* 33 (%)^a^	Other than White women *n =* 103 (%)^a^
**Antenatal (AN) care**			
**Number of women who received AN care**	***n =* 51 (85.0%)**	***n =* 30 (90.9%)**	***n =* 92(89.3%)**
Late first antenatal visit (>13 weeks)	14 (27.5)	9 (30)	20 (21.7)
Declined screening	10 (19.6)	2 (6.7)	19 (20.7)
Received recommended number of antenatal visits for gestation	42 (82.4)	24 (80)	82 (89.1)
Did Not Attend (DNA) two or more appointment(s)	11 (21.6)	3 (10)	15 (16.3)
% of total DNAs followed up	18/21 (85.7%)	10/11 (90.9%)	25/26 (96.2%)
**Postnatal (PN) care**			
**Number of women eligible for PN care in community**^b^	***n =* 33 (39.8%)**	***n =* 18 (21.7%)**	***n =* 32 (38.6%)**
0 home visits/appointments	1 (3.0)	0 (0)	0 (0)
1-2 home visits/appointments	4 (13.8)	1 (5.6)	10 (34.5)
3+ home visits/appointments	23 (79.3)	15 (83.3)	14 (48.3)
Unknown	5 (15.2)	2 (11.1)	8 (27.3)
**2 or more DNAs**	1 (3.4)	0 (0)	0 (0)
**% of total DNAs followed up**	0 (0)	N/A	N/A

a Due to rounding percentages may not add to 100%.

b Women who died after discharge from hospital and within the first year of giving birth.

### Mental health assessment

Overall, nearly three quarters (123/173, 71.1%) of women had evidence of antenatal routine mental health assessment, with little difference between the ethnic groups. While overall only 32⋅5% (27/83) had evidence of some kind of mental health assessment in the postnatal period, this was similar in the White British/Irish women and European/Other groups (5/33, 15⋅1% and 3/18, 16⋅7% respectively) and 59⋅4% (19/32) in Other than White groups. Assessment was done using the Whooley questions for 68⋅8% (119/173) of women in the antenatal period and 7⋅2% (6/83) of women in the postnatal period (see Supplementary Table S6).

### Unscheduled healthcare attendance

Nearly half of all women had three or more unscheduled attendances (outside of planned maternity care) at a healthcare service including General Practitioner, Accident and Emergency department, Day Assessment Unit/Triage (see Supplementary Table S7). Frequent attendance was 35⋅9% (37/103) in women from Other than White groups, 39⋅4% (13/33) in White European/Other and 60⋅0% (36/60) for White British/Irish women.

### Language needs

Of the whole cohort (196 women), 17 women had language needs (see Table 7); nearly a third of the White European/Other group (10/33, 30⋅3%). No woman from any cohort had a professional interpreter at all stages of maternity care. An interpreter was documented as being provided for 56⋅3% (9/16) of women at their first antenatal appointment, similar between the groups. An interpreter was provided for 69⋅2% (9/13) of women requiring intrapartum care: 100% (9/9) of White European/Other and 0% (0/4) women from Other than White groups.

**Table 7:** Maternity service language needs.

	WhiteBritish/Irish women*n =* 60 (%)^a^	European/Other women*n =* 33 (%)^a^	Other than White women*n =* 103 (%)^a^
Need for interpreter	0 (0)	10 (30.3)	7 (6.8)
**Provision of interpreter**			
First antenatal (booking) appointment	N/A	5/9 (55.6)	4/7 (57.1)
Antenatal care	N/A	3/8 (37.5)	4/7 (57.1)
Birth plan	N/A	1/7 (14.3)	0/5 (0)
Intrapartum	N/A	9/9 (100)	0/4 (0)
Postnatal visits	N/A	3/6 (50)	0/4 (0)
Appropriate provision	N/A	0 (0)	0 (0)

a Due to rounding percentages may not add to 100%.

### Barriers to following advice

Barriers to following advice from healthcare professionals were identified in a fifth of women (see Supplementary Table S8). These were mostly related to not taking prescribed medication, discharging themselves against medical advice and not attending specialist review. This was seen in a quarter (17/60, 28⋅3%) of White British/Irish women, 18⋅4% (19/103) of women from Other than White groups and 12⋅1% (4/33) of White European/Other women. The most common issue relating to not following advice among women from Other than White groups was not taking prescribed medication, while for White British/Irish women it was self-discharge against medical advice.

## Discussion

Accepting the limitations, the descriptive analysis from this study supports the hypothesis that differences in social risk factors or access and engagement with maternity services amongst women who died may not completely underlie ethnic disparities in maternal mortality in the UK.

The presence of three or more complex social factors was seen in a tenth (12/103, 11⋅7%) of Other than White women, and affected over a third (22/60, 36⋅7%) of White British/Irish women. While a quarter of all women booked at over 13 weeks gestation, the majority received the recommended antenatal visits appropriate for gestation. Routine mental health assessment was documented for most women antenatally, but fewer than a third postnatally. Overall, nearly half of the women had three or more unscheduled healthcare attendances. Unscheduled care is often associated with psychosocial and clinical needs that are not being met.^35^ Barriers to following advice from healthcare professionals were identified in a fifth of women. None of the 17 women who required an interpreter received provision throughout maternity care.

Three or more pre-existing physical co-morbidities were common in the Other than White groups (16/103, 15⋅5%) and over half had previous pregnancy problems (47/92, 51⋅1%). Two national case control studies have explored factors associated with maternal deaths in the UK. The first investigated the factors associated with maternal deaths from direct pregnancy complications and showed that medical comorbidities are importantly associated with direct obstetric deaths, as was being of Indian ethnicity.^36^ The second national case control study explored the risk factors associated with direct and indirect maternal death and identified medical comorbidities as well as smoking as being significant.^37^ This study also suggested that socio-economic inequalities were an important factor.

Our study is the first to attempt to systematically explore whether social risk factors or access and engagement with maternity services among women who died during or after pregnancy in the UK could underlie disparities in maternal mortality. For most groups, we included all available records of women who died. Where records were not available, these were largely for women who died from coincidental causes late in the postpartum year (e.g. malignancy) and whose deaths were less likely to be causally-related to pregnancy. The final sample is therefore likely to be representative of the overall group of women who died directly or indirectly related to pregnancy.

Due to researcher capacity, we sampled a computer-generated random number of White British/Irish women's medical records who died. Although this group formed the largest single ethnic group, this may limit representativeness. Methods for this review were robust, with two reviewers assessing and extracting data independently before discussion and agreement. Relatively few records (23/219, 10⋅5%) were unavailable for review; however, we did not numerate overall missing data due to the multiple fields in the data extraction form, which is a limitation of the study.

The categorisation of women from Other than White groups hides the heterogeneity of the numerous ethnic groups it contains and joining these groups together is said to be oversimplistic and to not allow exploration of their respective experiences.^38^ The study size is governed by the national numbers of women dying amongst those pregnant or giving birth; given that these numbers are small, the study would be underpowered for any formal statistical comparison and therefore no formal comparison has been carried out. We could not relate these findings to cause of death or quality of care as agreed by the MBBRACE UK assessors as data are no longer linked. While the data extraction tool was developed from a scoping review of the relevant literature and relevant NICE guidance for clinical care it is not possible to extract data for some barriers identified in the literature scope (such as culture and racism) through anonymised medical record review. In addition, social factors may not be documented in medical notes robustly, which may lead to an under-representation of these issues.

Evidence of the distribution of complex social factors in pregnancy by ethnicity is scarce and yet confidential enquiries into maternal death have consistently identified adverse impacts of complex social factors.^6^ We have identified that 55⋅3% (57/103) of women in the Other than White group had no complex social factors. However, multiple disadvantage (three or more complex social factors) was faced by a fifth of women. Though NICE guidance does not specifically address the breadth of social factors considered in this project, multi-agency plans to ensure coordinated care for women with complex social factors is recommended.^39^ Following identification, referral of complex social factors to other agencies for appropriate action and support was sometimes lacking and should be targeted for improvement.

Previous confidential enquiries have identified late first antenatal booking visit as a significant risk factor for maternal mortality,^40^ and this did not appear to be different between the groups. Evidence from research into women from Other than White groups who have not died has identified late booking as a particular issue.^8^^,^^15^ In our study, however, late booking occurred in 30⋅0% (9/30) of White European/Other women who died, 27⋅5% (14/60) of White British women and 21⋅7% (20/103) Other than White groups.

Communication difficulties are a key barrier to engaging with healthcare.^10^^,^^15^^,^^41^, ^42^, ^43^ Thus, NICE guidance recommends, for women with difficulty reading or speaking English, an interpreter should be provided who is not a member of the woman's family. While a need for an interpreter was only identified in 17 women in our study, a third of White European/Other women required interpreters. No woman received appropriate provision of language services throughout their care. A lack of available services has been noted across health and social sectors, not solely maternity care.^44^ International research also suggests that, in some instances, language services are available but usage by healthcare professionals is limited by a lack of training and time constraints.^45^, ^46^, ^47^ Rayment-Jones (2021) described difficulties for non-English speaking women with social risk factors accessing maternity services describing often having no choice of interpreter and being suspicious of both the confidentiality and quality of interpretation during appointments.^48^ This resulted in many preferring to use a trusted family member or friend to interpret for them. Barriers and facilitators to interpreter use need to be more clearly understood and solutions identified.

We identified barriers for a fifth of all women in following advice by healthcare professionals; nearly one in three for White British/Irish women. While in the majority of cases reviewed, healthcare workers worked hard to engage women, this warrants further exploration. There may be multiple reasons for disengagement including anxiety over potential harm to the fetus from adherence to drug therapy,^49^ not feeling safe or comfortable in healthcare environments,^50^ and not perceiving the care offered as useful.^51^

The reasons for the disparities in maternal mortality that exist in the UK are likely to be multifactorial and complex. A recent systematic review explored the maternal health inequalities encountered by Other than White women in the UK in relation to their experiences and use of services included a total of eight studies with various ethnicities and geographical locations.^52^ Five interconnected themes were identified following thematic analysis: communication, midwife-woman relationship, culture and social needs, bound together by healthcare services and systems. To effectively address all of these themes requires system level change which involves time, training and resources.

Further evidence of the complexity of the issue is provided by a recent UK national study which aimed to describe the women who died in the UK during or up to a year after the end of pregnancy, to compare the quality of care received by women from different aggregated ethnic groups, and to identify any structural or cultural biases or discrimination affecting their care.^53^ Results showed no differences in the proportionate causes of deaths during or up to a year after the end of pregnancy amongst women from different aggregated ethnic groups, nor were there any statistically significant differences in the assessed quality of care women received. Multiple areas of bias were identified in the care of a stratified random sample of 54 women received, with clinical, social and cultural complexity evident across all ethnic groups, as indeed our study identified. There was evidence of a lack of nuanced care which was most notable amongst women from Black aggregated ethnic groups who died and microaggressions were most prominent in the care of women from Asian aggregated ethnic groups who died.

The need to tackle these disparities in maternal death have been recognised by the Royal College of Obstetricians and Gynaecologists with the formation of the Race Equality Taskforce to better understand and tackle racial disparities in women's healthcare and racism within the obstetric and gynaecology workforce.^54^ Recent initiatives within the UK National Health Service (NHS) have been introduced in an attempt to tackle the issues identified. The NHS Long Term Plan set the aspiration that most women would receive continuity of carer aimed at reducing stillbirth, maternal and neonatal mortality and serious brain injury by 2025.^55^ In relation to the care of Other than White mothers, the review of *Better Births Four Years On* repeated the commitment made in the 2019 *NHS Long-term Plan* to improve maternity services by developing services for all pregnant women, targeting vulnerable groups including ‘BAME’ women, to receive continuity of care.^56^ This may go some way towards addressing the lack of nuanced care identified. More recently NHS England and NHS Improvement have developed *Equity and Equality Guidance for Local Maternity Systems* whereby plans will be set out to improve equity and equality, with financial support and monitoring of progress.^57^

While these initiatives offer opportunity for improvements which may address the inequalities in maternal mortality that exist there is evidence that the problem is broader than maternity services. A recent national cohort study found that socioeconomic and ethnic inequalities were responsible for a substantial proportion of stillbirths, preterm births and fetal growth restriction in England.^58^ The authors have proposed three key measures: targeting high risk groups with clinical interventions during pregnancy, such as nutrition programmes and improved access to high quality antenatal care; public health strategies to reduce inequalities in women's health before pregnancy; and comprehensive policies to tackle the fundamental causes of inequality, such as income, education, and employment, that indirectly influence pregnancy outcomes.

This study does not appear to identify differences in social risk factors or access and engagement with maternity services amongst women who died which might underlie ethnic disparities in maternal mortality. It appeared that White British/Irish women were more likely to attend unscheduled healthcare services and not follow recommended advice from healthcare professionals as well as be obese, smoke and experience ‘multiple social disadvantage’.

There remains an important need for further research to investigate why women from Other than White groups face significant disparities in maternal mortality risk in the UK, as the barriers to access and engagement studied were not predominantly found in this group. Areas of focus should be understanding the experiences of women and how best to address clinical, social and cultural complexity.

## Contributors

This study was undertaken over two years in part fulfilment of intercalated degrees in Public Health and Population Sciences at the University of Birmingham by EC and RN. EC developed the data extraction form and collected data from the women from Other than White groups, RN then collected data from the White women and analysed the whole cohort in the subsequent year, which was verified by FCS.

The study was designed by SK and EC/RN. The literature search was led by EC. The medical record reviews were led by EC and RN with FCS and SK acting as second researchers for validation of extracted data. Data were interpreted and the first draft of the manuscript written by EC and RN. SK supervised all aspects of the projects. MK contributed to the study design, data extraction tool and data interpretation. All authors had access to the Confidential Enquiry data, reviewed and critically revised the final paper for intellectual content and took the decision to submit for publication.

## Data sharing statement

Data may be requested through the Healthcare Quality Improvement Partnership https://www.hqip.org.uk/national-programmes/accessing-ncapop-data/.

## Declaration of interests

SK and MK are members of the MBRRACE-UK Collaboration.

MK reports grants from Heathcare Quality Improvement Partnership, National Institute for Health Research MRC and Wellbeing of Women; MK has an honorarium for Hooker Distinguished Visiting Professorship McMaster University.

SK reports a funded post from the University of Birmingham; is an NIHR grant holder, and is a collaborator on HQIP funded projects; was on the Steering Committee for NIHR funded trial- Impacted fetal head; was Chair on PreSePT Steering Committee of Health Foundation study; is Deputy Chair of HEE/NIHR Integrated Clinical Academic (ICA) Programme Pre-doctoral Clinical Academic Fellowship Scheme Panel.

RN declares that this work was started while she was undertaking an intercalated degree in Public Health and Population Sciences at the University of Birmingham. MBRRACE provided study materials – access to case notes.

EC declares that this work was started while she was undertaking an intercalated degree in Public Health and Population Sciences at the University of Birmingham.

FCS declares that this work was completed while employed as a research fellow at the University of Birmingham - funded by the National Institute for Health Research (NIHR) Applied Research Collaborative (ARC) West Midlands – Maternity Theme.

## Appendix Supplementary materials

Image, application 1
