# Supplementary material for: The role of social risk factors and engagement with maternity services in ethnic disparities in maternal mortality: A retrospective case note review
Source: eClinicalMedicine. 2022 Jul 29;52:101587. doi: 10.1016/j.eclinm.2022.101587 (PMC9340503; doi:10.1016/j.eclinm.2022.101587)
Supplement: Supplementary file 1 [file mmc1.docx]

Supplementary table 1: Literature search strategy

| 1 | (India* of Pakistan* or Bangladesh* or Asian).mp. |
| --- | --- |
| 2 | ("ethnic minority" or "minority ethnic" or "ethnic group" or "minority group").mp. |
| 3 | (BAME or Black or Black British or African or African American or Caribbean).mp. |
| 4 | 1 or 2 or 3 |
| 5 | exp Maternal Health Services/ |
| 6 | ((maternity or prenatal or postnatal or antenatal or perinatal or pregnan*) adj2 (care or service*)).mp. |
| 7 | 5 or 6 |
| 8 | 4 and 7 |
| 9 | exp United Kingdom/ |
| 10 | 8 and 9 |
| 11 | exp Health Services Accessibility/ |
| 12 | (access* or engage* or barrier* or limit* or disadvantage*).mp. |
| 13 | 11 or 12 |
| 14 | 10 and 13 |
| 15 | limit 14 to yr=”2010 -Current” |

Supplementary table 2: Study eligibility criteria

| **Inclusion Criteria** | **Exclusion Criteria** |
| --- | --- |
| Published during or after 2010 | Published prior to 2010 |
| English Language | Non-English Language studies |
| UK-based studies | Studies not based in the UK |
| Analysis or subgroup analysis of a Black or Asian ethnic minority group or a group with a Black or Asian majority | Studies that do not independently report outcomes for a Black or Asian ethnic group or a group with a Black or Asian majority |
| Outcome measure or findings relevant to access or engagement with maternity care services | Outcomes relevant only to general access to healthcare without specification of maternity services |
|  | Access and engagement with maternity services not included in outcomes or findings |

Supplementary table 3: Extracted data from literature search results

| **Study Title** | **Authors;**  **Journal;**  **Year of Publication** | **Study Design** | **Context and Setting** | **Outcomes** | **Methodology** | **Relevant Findings** |
| --- | --- | --- | --- | --- | --- | --- |
| Late booking amongst African women in a London borough, England: implications for health promotion. | Chinouya MJ; Madziva C.  Health Promotion International  2019 | Qualitative interviews | Interviews with 23 first generation Black African migrants who have used antenatal services in London from community settings | Cultural understandings of pregnancy and factors that influenced when to attend booking appointment. | Purposive and snowballing sampling. Topic guide for individual, semi-structured interviews. Interpretivist thematic analysis. | Four key themes: ‘unresolved immigration status’, ‘the importance of culture’, ‘lack of awareness’ and ‘cultural expressions of pregnancy’. Identified barriers include: cultural factors (e.g. inappropriate to disclose before 13 weeks), immigration status/barriers and lack of knowledge. |
| Maternal body mass index and access to antenatal care: a retrospective analysis of 619,502 births in England. | Barber C; Rankin J; Heslehurst N.  BMC Pregnancy & Childbirth  2017 | Secondary analysis of pre-existing data | National dataset of births in England 1989-2007 in 34 maternity units with information on BMI at booking and sociodemographics | Primary outcome = late access to antenatal care (gestational age at booking >13+6). Secondary outcome = trimester at booking. | Chi squared tests to compare BMI with trimester at booking. Adjusted analyses and individual association for socioeconomic factors. Binary logistic regression modelling. | All socioeconomic factors associated with late access and may be interrelated. Association with high BMI but greatest associations were for BME groups, young, unemployed and multiparous. Black/Black British had greatest effect size for all analyses. |
| Sociodemographic differences in women's experience of early labour care: a mixed methods study. | Henderson J; Redshaw M.  Obstetrics and Gynaecology  2017 | Secondary analysis of existing survey data | National maternity survey 2014 of ten thousand women 3 months postpartum randomly selected from birth registration statistics. Excluded those <16 or whose baby had died | Experiences of early labour care among women with different sociodemographic characteristics. Whether women who attend antenatal education were less worried about early labour and less likely to go to hospital early. | Questionnaire in 18 non-English languages with free return post envelope or online version.. Demographic data from ONS. Descriptive analysis, chi-squared, binary logistic regression for demographics, coding of free text. | Greater worry about knowing when labour would start and getting to hospital amongst BME women. BME less likely to attend classes due to not being offered or being booked up but BME women who did attend were no less likely to be worried. Free text showed feeling of neglect and dismissal. |
| Timing of the initiation of antenatal care: An exploratory qualitative study of women and service providers in East London. | Hatherall B; Morris J; Jamal F; Sweeney L; Wiggins M; Kaur I; Renton A; Harden A.  Midwifery.  2016 | Qualitative individual interviews and focus group discussions. | Pregnant and postnatal women and HCPs. Newham 2010/2011. Recruited through hospital-based maternity service, hospital based bilingual health advocacy service and community organisations | Factors influencing timing of initiation of antenatal booking. | 21 individual interviews with pregnant and postnatal women and 6 focus groups with 26 HCPs. Focus groups included 12 Bangladeshi, 13 Somali, 4 Lithuanian and 3 Polish women. Late and on time bookers included. Thematic analysis. | Difficulties navigating healthcare system, language barriers (registering, booking and travelling as well as communication with staff), lack of interpreters, competing priorities (housing, employment, education, care of other family/children), lack of information, perceived lack of value, GP access barriers. Mistrust and perception of handing over control. Some Somali and Bangladeshi women didn’t seek care if previous successful pregnancy, particularly if received little ant enatal care in last country. |
| Pregnancy as an ideal time for intervention to address the complex needs of black and minority ethnic women: views of British midwives. | Aquino MR; Edge D; Smith DM.  Midwifery.  2015 | Qualitative interviews. | One NHS Trust in Manchester within an area with high proportions of BME and deprivation over 2 months in 2013. | Midwives’ experiences of providing care for BME women. Midwives’ views on the relationship between maternal health inequalities and service delivery. | 20 semi-structured interviews using a topic guide. Maximum variation sampling strategy. Thematic analysis of data. | Need for unrestricted access to translation services. Lack of BME understanding of health system. Need for training to improve midwives’ cultural competence. Minority women have complex care needs. Need for better collaboration with other agencies outside of antenatal services. Lose information and difficult to assess understanding through interpreter. Interpreter requires more time but appointments standard length and limited access after hours. Lack of engagement may be due to lack of understanding of information about appointments. Cultural/religious beliefs not matched to services e.g. wanting female doctor or different practices. Complex needs for BME women inc. housing, welfare, long-term conditions, help to access benefits, complex immigration status. |
| Choosing motherhood: the complexities of pregnancy decision-making among young black women 'looked after' by the State. | Mantovani N; Thomas H.  Midwifery  2014 | Qualitative Interviews | Women aged 16-19 from Black minority ethnic groups, with a history of care, currently pregnant or a mother of a child <2 years old 2005-2007. | How interpersonal relationships affect the decision making process of an unexpected pregnancy. Experiences of health professionals during decision making. | Interpretative approach. Unstructured interviews. Purposive sampling for age, ethnicity, history of care and motherhood status. Thematic analysis. | Many had history of violence. Those who were raped before arriving in UK did not suspect their pregnancy. Receiving benefits, economic difficulties. Majority not with father. More recent migrants had no support network. Social isolation contributed to reduced health seeking. Perceived reaction of family members influenced decision making. Felt characterised and judged due to young age and economic situation. Lack of information and referral from GPs. Delay in social worker and counsellor allocation. Decision to keep/terminate pregnancy influenced by culture/religion. Health services didn’t always account for complex social factors in care given. Little evidence of needs assessment planning or multi-agency planning. HCPs had lack of updated knowledge about government policies on entitlements to care. |
| Experiencing maternity care: the care received and perceptions of women from different ethnic groups. | Henderson J; Gao H; Redshaw M.  BMC Pregnancy and Childbirth  2013 | Secondary analysis of survey data | Women aged 16 or over living in England who had recently given birth. 2010. | Use of maternity services. Experiences of care during pregnancy, labour and birth and the postnatal period. Demographic characteristic and comparisons. | Structured questionnaires of >50,000. 20 languages or option to complete via phone with interpreter. Logistic or multinomial regression models to assess associations with ethnicity. ORs and 95% CI for comparisons to white British. Adjustments for age, partner status, parity and mode of delivery. | Minority ethnic more likely to be younger, multiparous, without partner, less engaged with health services. Black African/Black Caribbean/Pakistani commence antenatal care later and fewer appointments and scans. Pakistani less likely to feel spoken to in a way they can understand, given help they need, sufficiently involved in decisions, or given choice about place of delivery. Poorer overall care rating by Pakistani. Black African more likely to have emergency CS. Lower confidence and trust in staff by Pakistani and Bangladeshi. Being left alone in labour and shortly after birth at a time when it worried them reported more amongst Asian and Black African. Poorer communication. Overall poorer rating of labour care. Black and Asian more likely to have longer postnatal hospital stay (may be linked to more CS/higher need for care). Bangladeshi, Black African and Pakistani less likely to have 5 or more postnatal visits or have postnatal health checks. Pakistani less likely to feel treated with kindness. Bangladeshi and Pakistani reported not enough information about emotional changes associated with childbirth. |
| Predictors of the timing of initiation of antenatal care in an ethnically diverse urban cohort in the UK. | Cresswell JA; Yu G; Hatherall B; Morris J; Jamal F; Harden A; Renton A.  BMC Pregnancy and Childbirth  2013 | Cross-sectional secondary analysis of routine data | Electronic patient record data from Newham University Hospital Trust 2008-2011 (20,135 women included). Area of high ethnic diversity and deprivation. | Late booking (after 12+6). Associated factors. Barriers and facilitators to early and consistent accesses to antenatal care. | Multivariable logistic regression with robust standard errors. | Late booking associated with non-British (White) ethnicity, non-English speaking and non-UK maternal birthplace. Most effects lost when adjusted for language and birth place suggesting language, culture and familiarity barriers. African/Caribbean still associated with late booking when English speaking and UK-born. Also associations with <20, high parity and temporary accommodation. Somali women highest proportion of late bookers. African highest magnitude of effects. Self-employed, semi-routine or routine and housewives more likely to book late. |
| Shared language is essential: communication in a multiethnic obstetric care setting. | Binder P; Borne Y; Johnsdotter S; Essen B.  Journal of Health Communication  2012 | Qualitative interviews and focus groups | Somali (39), Ghanaian (11) and White British (10) women living in Greater London. 62 Obstetric care providers from 5 hospitals. | Causes of communication barriers for optimal care of immigrant women. Expectations of maternity care by pregnant women from different ethnic background. Orientation of pregnant women towards staff of the same ethnic origin. | Snowballing through culture brokers to recruit most Somali women. Others purposive sampling through hospitals by head midwife or on call obstetrician. HCPs recruited through sign in maternity ward. Semi-structured individual interviews and focus groups with open-ended questions. Naturalistic enquiry analysis. | Language main problem for communication, particularly Somali. Interpreters important but concerns by providers about ability to understand medical terms in both languages and difficulty trusting interpreters. Accessibility and consistency of use of interpreters barrier. Time constraints issue. Issues identifying need for interpreter due to over-estimate of language skills or poorest language unaware of right to request/how. Issues with using family members with self-reported English skills or when breaking bad news. Discomfort sharing information with interpreters. Cultural and religious barriers. Those with language barrier had perceptions of being a “problem patient” and staff getting “fed up”. Lack of trust and noncompliance. Preference for female care providers perceived by staff but less so by interviewed women. |
| 'It's leaflet, leaflet, leaflet then, "see you later"': black Caribbean women's perceptions of perinatal mental health care. | Edge D.  British Journal of General Practice  2011 | Qualitative focus groups | Community settings in North West England. Sample of 42 Black Caribbean women drawn from larger study. 2007-2008. | Factors influencing low levels of consultation for perinatal depression amongst Black Caribbean women. Experiences of perinatal depression. Perceptions of ability of current services to meet needs. Components of ‘ideal service’ | Purposive sampling from larger study. 5 focus groups with 6-10 women. Interview guide with open questions. Framework thematic analysis. | Perceived lack of compassion by professionals. Inability to develop confiding relationships during pregnancy and childbirth. Lack of trust. Perception that staff too busy to address psychological needs. Thought that busy staff put infants needs above women. Lack of person-centred care and listening to what they want. Preferred alternative therapies and talking but thought GPs would just prescribe antidepressants. Thought that antidepressants could worsen rather than improve condition. Correlation between care experiences in pregnancy and labour and likelihood of seeking help postnatally. Particular barriers for sub-threshold mild/moderate. |
| Maternity services in multi-cultural Britain: using Q methodology to explore the views of first- and second-generation women of Pakistani origin. | Cross-Sudworth F; Williams A; Herron-Marx S.  Midwifery  2011 | Retrospective Q methodology | First or second generation Pakistani women 3-18 months postpartum from two Children’s centres in an inner city in the West Midlands | Maternity experiences and views of first- and second-generation Pakistani women. Inter-generational differences. Cultural needs of Pakistani women. | Analysis of semi-structured interviews, focus groups and literature review to produce themes. Reduction to statements reflecting overall content. Participants asked to sort response statements according to distribution grid representing level of agreement with statements. | No clear intergenerational differences. Those with less support and language barriers had greater needs. Trust and continuity of care important. Felt postnatal depression ignored. Inadequate support for non-English speakers. Educated felt more confident, well supported and better informed. Family and professional support important influencer of experiences. Lack of choice identified. Socially isolated had greater needs. Interpreter not always available and believed that speaking English improved care. Didn’t think that racism affected care. Some felt pressure to comply to traditions if lack of supportive family. |
| 'You need that loving tender care': maternity care experiences and expectations of ethnic minority women born in the United Kingdom. | Puthussery S; Twamley K; Macfarlane A; Harding S; Baron M.  Journal of Health Services Research & Policy  2010 | Qualitative interviews | 34 UK-born Black Caribbean, Black African, Indian Pakistani, Bangladeshi and Irish recent mothers from 9 NHS maternity units in England. | Maternity care experiences and expectations of UK-born ethnic minority women. | Interviewed at 3 months to 1 year after birth. Flexible topic guide used. NVivo qualitative analysis software. Grounded approach. Open coding and grouping into themes. | Being born in UK thought to remove language, system navigation and culture barriers. Empathy seen as most important part of care. Care thought to be unfriendly and dissatisfactory, particularly during postnatal period. Women with additional needs e.g. CS thought postnatal care inadequate. Black African and Black Caribbean expressed greatest dissatisfaction. Perception that staff busy, rushed and uninterested. Emotional support thought to be very important. Preference for continuity of care. Some made to feel like a ‘nuisance’ when asking for more information. Examples of poor communication causing worry or feeling unheard. Feeling of stress and tensions amongst staff. |
| Ethnic and social inequalities in women's experience of maternity care in England: results of a national survey. | Raleigh VS; Hussey D; Seccombe I; Hallt K.  Journal of the Royal Society of Medcine  2010 | Secondary analysis of survey data | Women aged >16 who had given birth. 2007 national Healthcare Commission survey of maternity services from records of 149 NHS acute trusts and two primary care trusts. | Inequalities in women’s experiences of maternity care during pregnancy, labour, birth and after birth between ethnicities, education, age and partner status. | Multiple logistic regression. Control for age, parity, self-reported disability, care trust, ethnic origin, partner status and education. Examination of associations of responses to ethnicity, partner status and education using ORs and 95% CIs. | Ethnic minority women more likely to access late. More likely to not have scan by 20 weeks. More complications in pregnancy and birth. Single more likely to access late, not have scan by 20 weeks, not attend antenatal classes, not have postnatal check-up, have complications. Educated more likely to access early, attend antenatal classes, breastfeed, have postnatal check-up and less likely to have complications. Students received late care. Black/Asian less likely to say they had a choice about birth place. Black less likely to say they had midwife contact details. Ethnic minority less likely to trust in staff. Asian and Black more likely to have planned Caesarean. Ethnic minorities less likely to have seen midwife as much as wanted. |
| Falling through the net – black and minority ethnic women and perinatal mental healthcare: health professionals' views. | Edge D.  General Hospital Psychiatry  2010 | Qualitative interviews and focus groups. | HCPs from the North of England 2007-2008. Recruited from antenatal community clinics, a large teaching hospital, GP and a specialist voluntary sector agency | Healthcare practitioners’ experiences of and views on managing perinatal depression among Black Caribbean women. | Purposive sample. Topic guide from literature. NVivo qualitative analysis software used. Framework analysis. | Inadequate training and lack of confidence for identifying specific needs of Black women and managing perinatal depression more generally, particularly mild/mod subthreshold. Failure to screen routinely, confusion about professional roles and poorly defined care pathways causing women to ‘fall through the net’. Antenatal depression not treated or used to identify risk of postnatal depression. Physical conditions thought to be more important. Time and resource issues barrier to addressing perinatal depression. Screening tools thought to be ineffective. Black women perceived to be particularly adverse to health surveillance. Sociocultural barriers and lack of cultural competence. Lone parents lacked support. |
| Ethnic differences in risk factors for adverse birth outcomes between Pakistani, Bangladeshi, and White British mothers. | Garcia, R; Ali, N; Guppy, A; Griffiths, M; Randhawa, G  Journal of Advanced Nursing  2020 | Retrospective secondary analysis of routine data | Data from one hospital in Luton 2008-2013 for White British, Bangladeshi and Pakistani women. | Presence of risk factors for poor pregnancy outcomes. Pregnancy behaviours. Distribution of risk factors and health behaviours during pregnancy by ethnicity. | Part of a wider mixed-methods study. Data from Ciconia Maternity Information System. Cross tabulations, ANCOVA, adjusted standardised residuals and Pearson’s chi squared. | Pakistani women had highest number of risk factors. Diabetes common amongst Pakistani and Bangladeshi and very high for Pakistani. Pakistani and Bangladeshi more likely to book late. White British less likely to be multiparous. Pakistani mothers more likely to have BMI<18. Bangladeshi more likely to use insulin. |
| The obstetric care of asylum seekers and refugee women in the UK. | Asif, S; Baugh, A; Jones, N  Obstetrics & Gynaecology  2015 | Literature review | International literature regarding migrants and asylum seekers applied to a UK setting. | Medical, sexual and psychosocial issues affecting pregnant asylum seekers and refugee women in the UK. | Review of Literature and current regulations. Methods not given. | Late booking. Lack of interpreters. Stress and uncertainty for those applying for asylum. Newly arrived at greatest risk. Poverty and transport issues causing missed appointments. Single parents and postnatal depression causing disengagement with healthcare services. Particularly vulnerable when arriving in new country and pregnant as a result of sexual assault. Asylum seekers unable to work or claim benefits. Poor accommodation for asylum seekers and moved around a lot. Poor nutrition, infectious diseases and mental health issues common amongst migrants. Undiagnosed chronic diseases, poor oral hygiene and previous injury and trauma. History of FGM amongst women from Africa may have complications. Language, culture and family support network. Pre-existing HIV/TB can increase risk of poor outcomes. Studies show higher rates of Caesarean for Somali and Asian women. Migrant women twice as likely to develop postnatal depression. Issues with using interpreters and with using family members. Late arrival during pregnancy with lack of medical records and undiagnosed conditions. Evidence of perceived racism and rudeness from staff. |

Supplementary table 4: Summary of literature findings

| Key barriers identified | Details of findings | Studies |
| --- | --- | --- |
| Language | - Barrier to registering, booking and travelling to appointments as well as communication with staff | Hatherall, 2016; Aquino, 2015; Cresswell, 2013; Binder, 2012; Cross-Sudworth, 2011; Asif, 2015 |
| Issues with interpreters | - Lack/poor use of interpreters - *Caveats* to using interpreters - Issues with using family members | Hatherall, 2016; Aquino, 2015; Binder, 2012; Cross-Sudworth, 2011; Asif, 2015 |
| Culture | - Cultural health beliefs influenced late booking and decision making - Need for improved cultural competence of services | Chinouya, 2019; Hatherall, 2016; Aquino, 2015; Cresswell, 2013; Binder, 2012; Cross-Sudworth, 2011; Edge, 2010; Asif, 2015 |
| Late booking | - Late booking associated with Black and Asian women, non-English speaking and non-UK birthplace - Previous experiences and social and demographic contributors | Barber, 2017; Hatherall, 2016;  Mantovani, 2014; Henderson, 2013; Cresswell, 2013; Raleigh, 2010; Garcia, 2020; Asif, 2015 |
| Fewer appointments and less engagement | - Ethnicity, demographic and social factors | Henderson, 2013; Raleigh, 2010; Asif, 2015 |
| Immigration status | - Unresolved immigration status contributed to late booking - Lack of updated knowledge about entitlements to care from HCPs - Being UK-born thought to remove language, culture and navigation barriers - Newly arrived face greatest barriers - Asylum seekers unable to work or claim benefits and may have housing issues or be moved | Chinouya, 2019; Aquino, 2015; Puthussery, 2010; Asif, 2015 |
| Dissatisfaction with care | - Lower satisfaction with overall care for minority ethnic women - Lack of compassion and choice - Perception of staff being too busy and feeling like a ‘nuisance’ - Perceived racism and rudeness | Henderson, 2017; Henderson, 2013; Binder, 2012; Edge, 2011; Puthussery, 2010; Raleigh, 2010; Asif, 2015 |
| Lack of knowledge | - Difficulty accessing and navigating healthcare system - Lack of knowledge about benefits of care | Chinouya, 2019; Hatherall, 2016; Aquino, 2015 |
| Complex social needs | - Housing, employment, education, care responsibilities, welfare, financial issues, domestic violence, lack of support, raised BMI and young linked to reduced access - Poor coordination with other health and care agencies to address social needs | Hatherall, 2016; Aquino, 2015; Mantovani, 2014; Cross-Sudworth, 2011; Asif, 2015; Barber, 2017; Henderson, 2017, Cresswell 2013; Raleigh 2010. |
| Physical health needs | - Differing and greater medical needs - More pregnancy complications - Undiagnosed chronic conditions | Aquino 2015; Henderson 2013; Raleigh 2010; Garcia 2020; Asif 2015 |
| Mental health needs | - Greater welfare needs - Mental wellbeing and perinatal depression neglected | Aquino, 2015; Henderson, 2013; Edge, 2011; Cross-Sudworth, 2011; Edge, 2010; Asif, 2015 |

Supplementary table 5: Final standardised data extraction form for review of casenotes

Language

Complex social factors

Demographics

| Case ID | |  | | | | | | |
| --- | --- | --- | --- | --- | --- | --- | --- | --- |
| Cause and place of death  *Not used in paper* | |  | | | | | | |
| Days gestation/postnatal at death | |  | | | | | | |
| Summary of case and care | |  | | | | | | |
| No maternity service involvement | | Yes/No | | | | | | |
|  |  |  | | | | | | |
|  | Ethnicity |  | | | | | | |
|  | Age *at death* |  | | | | | | |
|  | BMI *at booking* |  | | | | | | |
|  | Country of birth |  | | | | | | |
|  | Citizenship status |  | | | | | | |
|  | Payment for services |  | | | | | | |
|  | Employment status |  | | | | | | |
|  | Partner status/support |  | | | | | | |
|  | *If not documented assumed to be No* | Present (yes/no) | | Identified  *By maternity services* | | | Actions taken | |
|  | Significant financial need |  | |  | | |  | |
|  | Insecure housing |  | |  | | |  | |
|  | Substance misuse |  | |  | | |  | |
|  | Smoking |  | |  | | |  | |
|  | Criminal justice |  | |  | | |  | |
|  | Social services involvement |  | |  | | |  | |
|  | Learning/physical disability |  | |  | | |  | |
|  | Domestic abuse |  | |  | | |  | |
|  | Mental health issues |  | |  | | |  | |
|  | Young (<20yrs) |  | |  | | |  | |
|  | Recent migrant (<1yr) |  | |  | | |  | |
|  | Need for interpreter |  | | | | | | |
|  |  | Present | | Type of interpretation | | | | |
|  | Booking |  | |  | | | | |
|  | Antenatally (number) |  | |  | | | | |
|  | Birth plan |  | |  | | | | |
|  | Intrapartum |  | |  | | | | |
|  | Postnatally (number) |  | |  | | | | |
| Antenatal | Gravity and Parity |  | | | | | | |
|  | Late booking (>13 weeks) | Yes/No | | Reason if late | | | | |
|  |  |  | |  | | | | |
|  | Routine antenatal visits (Number) |  | | | | | | |
|  | DNAs | Number | | Reasons | | | Follow-up | |
|  |  |  | |  | | |  | |
|  | Declined screening | Type of screening | | Reason missed | | | | |
|  |  |  | |  | | | | |
|  | Emotional assessment | None evidenced | | Whooley questions | No concerns tick-box | | | Action taken |
|  |  |  | |  |  | | |  |
|  | Pregnancy related comorbidities | Present | | Identified | | Action | | |
|  |  |  | |  | |  | | |
|  | Other existing physical /mental health needs |  | |  | |  | | |
|  | History of FGM | Present (yes/no) | | | Type and referral | | | |
|  |  |  | | |  | | | |
|  | Other antenatal attendances |  | Number | Reasons for attendance | | | | |
|  |  | GP |  |  | | | | |
|  |  | Triage/DAU/A&E |  |  | | | | |
|  |  | Hospital admissions |  |  | | | | |
| Intrapartum | No intrapartum care | Yes/No | | | | | | |
|  |  |  | | | | | | |
|  | Mode & place of delivery |  | | | | | | |
|  | Delivery summary |  | | | | | | |
|  | No postnatal care | Yes/No | | | | | | |
|  |  |  | | | | | | |
| Postnatal | Number of postnatal visits |  | | | | | | |
|  | DNAs | Number | | Reasons | | Follow up | | |
|  |  |  | |  | |  | | |
|  | Emotional assessment | None evidenced | | Whooley questions | No concerns tick-box | | | Action |
|  |  |  | |  |  | | |  |
| Requests made for female HCP | |  | | | | | | |
| Barriers to Adherence | | Yes/No | | Medication | Self-discharge | | | Other |
|  |  |  | |  |  | | |  |
| Evidence of MDT working (details) | |  | | | | | | |
| Anxiety expressed by mum | |  | | | | | | |
| Transfers between trusts | |  | | | | | | |
| Evidence of racism/discrimination | |  | | | | | | |
| Local review carried out | | Yes/No | | Who by | | | | |
|  |  |  | |  | | | | |
| Local review mentioned but not evidenced | | Yes/No | | | | | | |
|  |  |  | | | | | | |
| Additional Comments | |  | | | | | | |

Supplementary table 6: Mental health assessment

| **Antenatal** (excluding those not booked for care with maternity services) | **White**  **British / Irish** **women**  n=51 (%) | **White European / Other women**  n=30 (%) | **Other than White women**  n=92 (%) |
| --- | --- | --- | --- |
| None evidenced | 14 (27.5%) | 9 (30%) | 27 (29.3%) |
| Whooley questions completed | 37 (72.5%) | 20 (66.7%) | 62 (67.4%) |
| No concerns tick box | 0 | 1 (3.3%) | 0 |
| Other | 0 | 0 | 3 (3.3%) |
| **Number of women with an antenatal mental health assessment** | **37 (72.5%)** | **21 (70.0%)** | **65 (70.7%)** |
| **Postnatal** (excluding those without postnatal care) | **n=33 (%)** | **n=18 (%)** | **n=32 (%)** |
| None evidenced | 28 (84.8%) | 15 (83.3%) | 13 (40.6%) |
| Whooley questions | 1 (3%) | 0 | 5 (15.6%) |
| No concerns tick-box | 1 (3%) | 1 (5.6%) | 10 (31.3%) |
| Other | 3 (9.1%) | 2 (11.1%) | 4 (12.5%) |
| **Number of women with a postnatal mental health assessment** | **5 (15.1%)** | **3 (16.7%)** | **19 (59.4%)** |

Supplementary table 7: Unscheduled healthcare attendances

| **Three or more attendances at each of the following:** | **White**  **British / Irish** **women** n=60 (%) | **White European / Other** **women**  n=33 (%) | **Other than White women**  n=103 (%) |
| --- | --- | --- | --- |
| General Practitioner (GP) | 20 (33.3%) | 9 (27.3%) | 20 (19.4%) |
| Accident & Emergency (A+E) | 3 (5%) | 1 (3%) | 1 (1%) |
| Day Assessment Unit (DAU) / Triage | 13 (21.7%) | 6 (18.2%) | 7 (6.8%) |
| Total number of women with 3 or more combined GP/A+E/DAU/Triage attendances | 36 (60%) | 13 (39.4%) | 37 (35.9%) |

Supplementary table 8: Barriers to following advice from healthcare professionals

|  | **White**  **British / Irish** **women**  n=60 (%) | **White European / Other** **women**  n=33 (%) | **Other than White women**  n=103 (%) |
| --- | --- | --- | --- |
| Number of women who had barrier(s) to following advice | 17 (28.3%) | 4 (12.1%) | 19 (18.4%) |
| **Types of barriers** | | | |
| Not taking prescribed medications | 3 (5%) | 1 (3%) | 11 (10.7%) |
| Self-discharge against medical advice | 8 (13.3%) | 2 (6.1%) | 10 (9.7%) |
| Not attending specialist review | 4 (6.7%) | 1 (3%) | 0 |
| Other | 3 (5%) | 0 | 0 |

Supplementary Figure 1: Prisma flow diagram of literature search results

Records identified through MEDLINE and CINAHL searches

(n=55)

Articles unable to access

(n=2)

Records removed

(n=22)

Reasons for exclusion:

- Population not black or Asian or no black or Asian subgroup analysis
- Outcomes not relevant to access and engagement with maternity services

Full texts reviewed

(n=16)

Full texts screened

(n=18)

Abstracts screened

(n=40)

Records after duplicates removed

(n=40)
